# Quantification of pulmonary arterial pressure with 4D flow cardiac MRI velocity mapping in patients with suspected pulmonary hypertension: Comparison with right heart catheterization

**DOI:** 10.1371/journal.pone.0346600

**Published:** 2026-04-23

**Authors:** Charlotte Dantoing, Roger Bouzerar, Yohann Bohbot, Isabelle Mayeux, Raphaël Pichois, Cédric Renard

**Affiliations:** 1 Department of Radiology, Amiens University Hospital, Amiens, France; 2 Biophysics and Image Processing Unit, Amiens University Hospital, Amiens, France; 3 Department of Cardiology, Amiens University Hospital, Amiens, France; 4 Department of Pneumology, Amiens University Hospital, Amiens, France; Showa University: Showa Daigaku, JAPAN

## Abstract

**Objectives:**

4D flow MRI is becoming a promising tool to assess pulmonary hypertension which remains a progressive fatal disease. The aim of this study was to compare the quantification of pulmonary arterial pressure derived from 4D flow MRI with right heart catheterization in patients with pulmonary hypertension.

**Methods:**

Thirty-two patients (22 men, 10 women, mean age 62.6 years old) with known or suspected pulmonary hypertension were enrolled in this prospective study. Subjects were split into two consecutive groups, with the first 22 subjects dedicated to analysis and the last 10 subjects dedicated to validation. All patients underwent right heart catheterization and cardiac MRI examinations. Pulmonary arterial pressures were measured by catheterization. An accelerated kat-arc 4D flow MRI sequence allowed the analysis of cardiac blood and pulmonary artery (PA) flows. Multivariate linear regression models were obtained using stepwise, bottom-up and top-down covariate selection procedures.

**Results:**

Using right heart catheterization as reference, the multivariate estimates of mean (mPAP) and systolic (sPAP) pulmonary arterial pressures only included 4D flow MRI parameters: mean helicity in right ventricle (RV), mean vorticity in right atrium (RA) and maximum cross-sectional PA area (A_max__PA). The models yielded mPAP = 0.04.A_max__PA + 0.061.mean_helicity_RV – 2.42 (R² = 0.69) and sPAP = 0.066.A_max__PA + 0.134.mean_helicity_RV – 0.613.mean_vorticity_RA + 23.98 (R² = 0.80). Bland-Altman bias were 0.42 and 0.38 mmHg, respectively.

**Conclusion:**

This study suggests that kat-arc accelerated 4D flow MRI is a potential non-invasive technique for pulmonary arterial pressure estimation. Therefore, this short-duration sequence could become a useful diagnostic and follow-up exam for patients with pulmonary hypertension.

## Introduction

Pulmonary hypertension (PH) is a severe and multifactorial disease characterized by a progressive increase in mean pulmonary arterial pressure (mPAP) and pulmonary vascular resistance (PVR). It is defined as an mPAP exceeding 20 mm Hg at rest [1], measured invasively by right heart catheterization (RHC) [2,3]. Based on the combination of RHC parameters, underlying etiology, clinical presentation and response to treatment, the current system of classification identifies five categories of PH [2,4]. PH is a complication of various cardiovascular and pulmonary diseases and is associated with increased morbidity and mortality attributed to right heart failure resulting from the increased afterload secondary to elevated pulmonary arterial pressures [5]. The right ventricle (RV) compensates for the increased afterload with remodeling: enlargement and hypertrophy. The evolution of this disease is also characterized by tricuspid and pulmonary valvular insufficiencies.

Diagnostic of PH is confirmed during RHC which is considered as the gold standard in spite of its invasive nature [6].

Trans-thoracic echocardiography (TTE) is the non-invasive screening technique for systolic pulmonary arterial pressure (sPAP) estimation, for assessment of cardiac function and to rule out secondary causes of pulmonary hypertension such as left heart disease or congenital heart disease [2,7]. The approximation of sPAP is derived from the simplified Bernoulli equation using the maximal velocity of the tricuspid valve regurgitation (V_max_ TVR) and an estimate of the right atrial pressure (RAP) [8,9]. However, TTE derived pulmonary pressures has specific limitations: poor acoustic window, user dependency, variability of right atrial pressure and controversial data regarding pulmonary hemodynamics [10,11].

Cardiac MRI is performed to assess cardiac function, myocardial morphology and viability for prognosis and severity evaluation in PH [2,12,13]. MRI is a non-invasive and non-ionizing technique providing good temporal and spatial resolution and demonstrating better reproducibility than TTE for estimating ventricular parameters [14]. Several morphological changes such as RV dilation and hypertrophy, myocardial septal fibrosis or dilation of the pulmonary artery have been reported in PH [12,15]. Functional flow abnormalities such as tricuspid and pulmonary valvular regurgitation or decrease in cardiac output have also been noticed. Cardiovascular blood flows can be assessed using 2D or 4D MR phase-contrast sequences. A recent study demonstrated the accuracy of a 4D flow MR sequence in measuring systemic and pulmonary blood flows in patients with PAH associated with congenital heart disease, when compared with RHC flow measurements [16]. A few studies have shown that 4D flow MRI measurements can be correlated with mPAP and PVR [7,17–19]. Furthermore, vortical structures can be observed during some phases of the cardiac cycle in PH subjects and the visual duration of the vortex during the cardiac cycle appeared to correlate with elevated mPAP [7,17,20,21]. Nevertheless, this finding has not always been substantiated [22]. In order to describe and quantify these vortices, the vorticity vector, defined as the curl of the flow velocity, is calculated locally from the velocity field. Several studies have shown that vorticity is correlated with mPAP and PVR measured during right heart catheterization [18,23]. Another useful quantitative parameter for the characterization of vortices is the so called “helicity” (H), defined as the scalar product between the local velocity and vorticity vectors [24]. This parameter allows to classify the observed vortex as longitudinal or transverse with respect to the flow streamlines.

In the literature, the number of studies regarding vorticity and helicity in PH is limited and further work is needed to support the diagnostic value of 4D flow MRI in PH and to establish models for non-invasive mPAP estimation. The robustness of flow measurements and cardiac volumes could potentially allow the use of 4D flow MRI as a unique diagnostic, prognostic and follow-up procedure in PH.

The aim of this study was to propose an estimate of the pulmonary artery pressure based on the hemodynamic parameters derived from an accelerated kat-ARC 4D flow sequence compared with the results of right heart catheterization in patients with or suspected of having PH.

## Methods

### Study population

Patients who underwent RHC and cardiac MRI for PH (diagnostic or follow-up) were enrolled from September 22, 2020 to August 26, 2022. This prospective single-center study was approved by the Committee for the Protection of Individuals CPP EST IV (Strasbourg, France) on July 29, 2020 (Approval number: 2020-A01643-36), and complied with the declaration of Helsinki. Written informed consent requirement was waived, but a few weeks before the examinations, an information sheet offering the patients to participate in the study, and approved by the Ethics Committee, was sent. The need for written informed consent was waived as all procedures were performed as part of clinical care. Oral informed consent was obtained from the participants to the study and recorded in the patient’s medical file.

Inclusion criteria were: adult patient with or suspected of having PH, patient who undergo RHC and an MRI exam scheduled within a maximum of 15 days from the date of RHC. Exclusion criteria were: change in medical treatment between RHC and MRI measurements, age < 18 years, severe obesity (BMI > 35 kg/m²), pregnancy, all other contra-indications to MRI or contrast agent.

Patients were divided into two consecutive groups: the first 22 were dedicated to the analysis and the last 10 patients were dedicated to the validation.

### Catheterization

All patients underwent a RHC by a referent pulmonologist (15 years of experience). RHC was performed with a Swan-Ganz catheter using a transjugular approach. Measurements included mean pulmonary arterial pressure (mPAP), systolic pulmonary arterial pressure (sPAP), diastolic pulmonary arterial pressure (dPAP), pulmonary artery wedge pressure (PAWP), right atrial pressure (RAP), thermodilution cardiac output (CO), cardiac index (CI), as well as the pulmonary vascular resistance (PVR). Pulmonary hypertension was defined as RHC-mPAP ≥ 20 mmHg at rest [1].

### MRI

MR imaging was performed at 1.5T (Optima MR450W, GE Healthcare, Milwaukee, WI) using an 8-element cardiac array coil (C-Body 30 Small) with the patient in supine position. The standard MRI protocol included ECG-gated 2D-cine SSFP sequences in the standard cardiac views (4-chamber views, right and left ventricular 2-chamber views, and short-axis planes from base to apex), a pulmonary 3D MR angiographic sequence and a late enhancement sequence in the short axis plane of the ventricles, performed at least 10 minutes after contrast injection [25]. A 4D flow kat-ARC accelerated sequence [26,27] in free-breathing was added to the usual protocol between MR pulmonary angiography and the LGE sequences. It did not significantly increase examination time. Its duration was around 6 minutes, during the waiting period before LGE imaging. This axial acquisition was performed with an exploration volume box, including 150 slices, positioned on the cardiac mass (above the aortic arch to the inferior wall of the heart) using Venc = 400 cm/s in the three directions. The main parameters of the 4D flow MRI sequence are summarized in Table 1.

**Table 1:** Imaging parameters of the 4D flow MRI sequence.

Parameters	Values
Synchronization	ECG
Breathing	Free breathing
TR (ms)	4.3
TE (ms)	1.9
Flip angle (°)	14
In-plane resolution (mm)	2.2 x 2.2
Slice thickness (mm)	2.4 (zip 1.2)
Time resolution (ms)	63
Acceleration method	kat-ARC
Phases per cardiac cycle	20
Total duration (min)	5

Note: MRI magnetic resonance imaging, TR repetition time, TE echo time.

### Data processing

Cardiac MRI data were analyzed by a junior radiologist under the supervision of an experienced radiologist (15 years of experience in cardiac imaging).

The morphological series were processed using a commercially available software (CMR42 version 5.9, Circle Cardiovascular Imaging, Calgary, Canada) dedicated to the interpretation of cardiac MRI. The following measurements were performed from the short-axis series: LV and RV end-diastolic diameters (LVEDD, RVEDD), end-diastolic (LVEDV, RVEDV) and end-systolic (LVESV, RVESV) ventricular volumes indexed to the body surface area, as well as ventricular ejection fractions (LVEF and RVEF) assessed from a modified Simpson’s method.

The LV/RV diameter ratio was measured in an end-diastolic 4-chamber cine view. In addition, RV myocardial mass and right atrial surface area, measured on 4-chamber cine images at end-systole phase, were also assessed. Inversion of the interventricular septum and septal fibrosis characterized by predominantly nodular LGE in anterior and posterior RV insertion site to interventricular septum zones were recorded [13].

**Fig 1:**
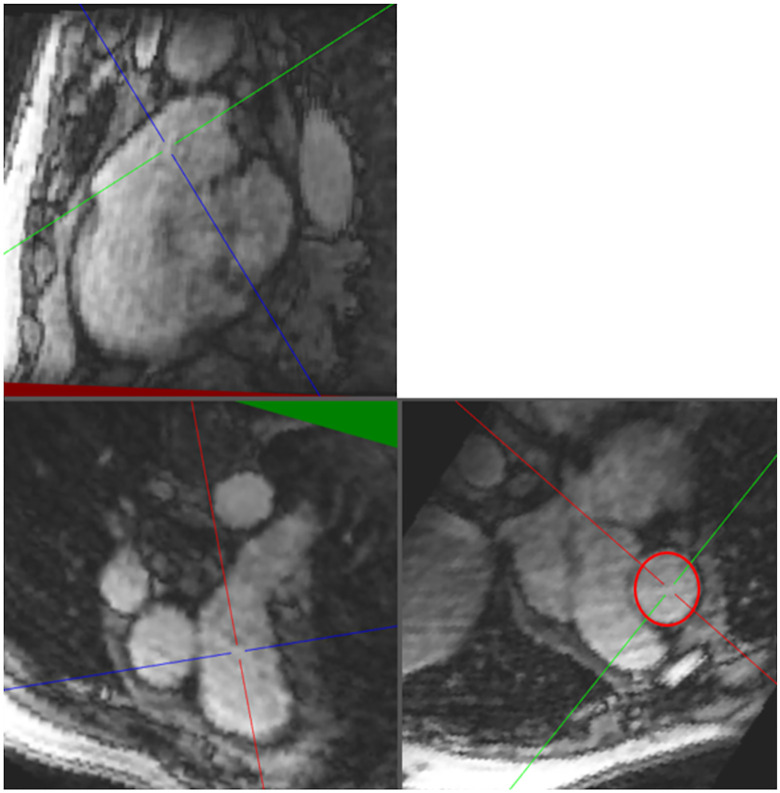
Placement of the double oblique axes to obtain the PA measurement plane.

Post-processing of 4D flow images were performed using a dedicated in-house software specifically written in C++ language. This visualization and analysis software is based upon Qt (Qt Company Ltd) for the user interface and VTK/ITK libraries (Kitware Inc) for 2D/3D rendering. After importation of the DICOM series, a semi-automatic correction of phase offset, using 2D third order polynomial interpolation, was performed prior to processing. For further detail about the implementation, see Supplementary Data 1. Slice planes were placed using double oblique axis reformatting, at the time of interpretation. Streamlines representation of velocity vectors was used to assist in correct positioning. A first slice plane was placed at the level of the pulmonary artery trunk, perpendicular to the vessel wall, between the plane of the valve annulus and the pulmonary bifurcation, approximately 1 cm downstream of the valve (Fig 1).

**Fig 2:**
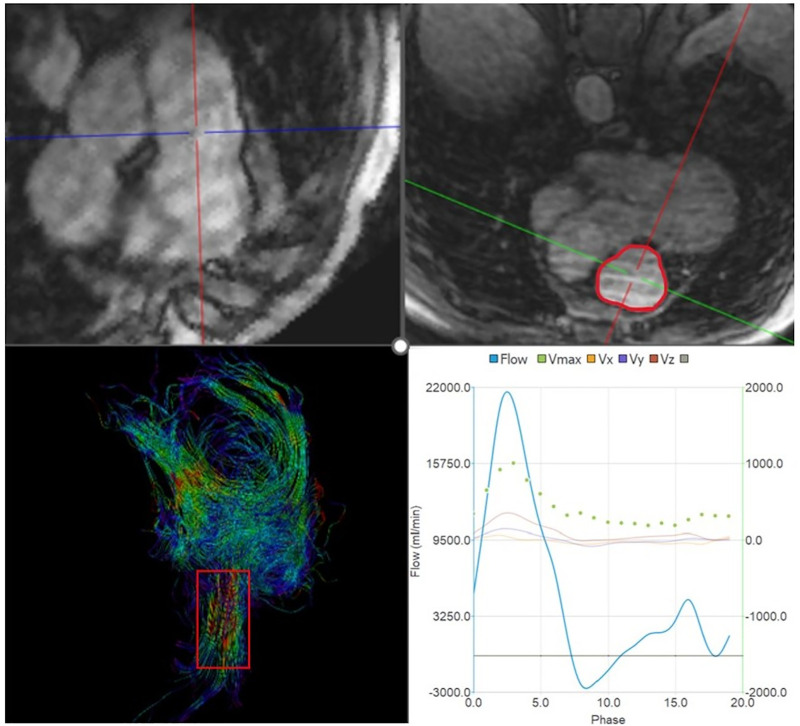
Placement of the plane at the level of the pulmonary valve (top). After contouring, visualization of the streamlines during diastole showing a narrowing due to the valve leakage (bottom).

The pulmonary artery was then contoured on the resulting plane through all phases of the cardiac cycle in order to measure the maximum systolic pulmonary artery ejection velocity (V_max__PA), the maximum systolic and minimum diastolic PA areas and the relative change in the PA cross-sectional area. A second plane was placed parallel to the valvular plane at the level of the pulmonary valve, and streamlines were displayed for each phase of the diastolic period. Pulmonary regurgitation could therefore be visualized by a narrowing of the flow at and upstream of the valve with the highest velocities being encoded in red (Fig 2).

The maximum velocity was therefore automatically obtained from this area. The third plane was placed through the right ventricle, parallel and close to the tricuspid valve plane. Tricuspid regurgitation was also visualized by flow narrowing at the valve plane and in the RA. The vorticity magnitudes were computed from the whole 3D velocity field and then measured in PA, RA and RV (see S1 File). At the PA level, the systolic streamlines were segmented by manually positioning a cutting plane before the pulmonary bifurcation; the median and 90th percentile values were then measured along the streamlines.

Following a protocol similar to Hirtler et al [28], vorticity and helicity were assessed in the RA and RV regions identified on a conventional 4-chamber view derived from the magnitude volume. Two regions of interest encompassing these structures near their boundaries were manually delineated, and the maximum and average values over all phases of the cardiac cycle were subsequently extracted. The maximum and mean values across the cardiac cycle were then calculated and reported.

The RV/LV diameter ratio was also measured in a MPR-reconstructed 4-chamber magnitude image.

### Statistical analysis

Statistical analysis was performed using IBM SPSS Statistics 28.0. Descriptive statistics were calculated for all variables of interest. The significance level was set at 5%. The Spearman correlation coefficient was calculated to test the correlation between quantitative variables.

Multivariate linear regression models were obtained using different covariate selection procedures: stepwise bottom-up and top-down procedures. The variables to be tested were selected statistically and according to their clinical relevance: Septal fibrosis, Inversion of the interventricular septum, RVEF, LVEF, RV mass, RVEDV, RVESV, RVEDD, RA surface, RV/LV diameters ratio, V_max__PA, V_max_PVR, V_max_TVR, minimum & maximum PA cross-sectional areas, PA Vorticity at 50^th^ & 90^th^ percentile, RV and RA mean and maximum Vorticity, RV and RA mean and maximum Helicity.

Multicollinearity of variables was tested by the variance inflation factor (VIF < 5). The Durbin-Watson test for independence of residuals was also performed, and homoscedasticity of residuals was graphically checked. Bland-Altman analysis was used to evaluate the agreement between pressure estimations. Normality of the differences between methods was assessed using the Shapiro–Wilk test. As the distribution of the differences deviated from normality, a bootstrap-based Bland–Altman analysis [29,30] was performed to obtain robust estimates of the mean bias and limits of agreement of all our Bland-Altman analyses. Bootstrap resampling with 10 000 replications was used to derive 95% confidence intervals for the bias and limits of agreement without relying on the normality assumption. A Wilcoxon test was used to compare the biases to zero.

Validation of the models was performed using PRESS (Prediction Sum-Of-Squares) analysis (package qpcR), which is a surrogate measure of cross-validation for small sample sizes [31]. This algorithm is a leave-one-out refitting and prediction method that returns the Press R-squared value, equivalent to R-square. In addition, a Bland-Altman analysis was performed using an independent validation set of patients.

## Results

Twenty-two patients (17 men) and ten patients (5 men) were included in the analysis and validation group, respectively. The baseline characteristics of these cohorts are summarized in Table 2.

**Table 2:** Baseline characteristics of the two groups of patients.

Characteristics	Analysis group (n = 22)	Validation group (n = 10)
Sex (M/F) – n (%)	17/5 (77.3/ 22.7)	5/5 (50.0/ 50.0)
Age	61.2 ± 15.8	65.9 ± 11.0
BMI (kg/m²)	26.4 ± 5.0	29.4 ± 6.7
Heart rate (bpm)	77.6 ± 12.9	71.9 ± 13.0
Systolic BP (mmHg)	120.9 ± 17.7	134.0 ± 16.3
Diastolic BP (mmHg)	72.0 ± 10.4	76.1 ± 12.4
Mean BP (mmHg)	86.8 ± 14.2	91.3 ± 12.6
Time between MRI and catheterization (day)	3 [3; 3]	3 [3; 3]

Note: *Values expressed as mean ± standard deviation or median (interquartile range)*, M/F: Male/Female, BMI: Body Mass Index = Weight/(Height)², BP: Blood Pressure, MRI magnetic resonance imaging.

### Analysis group

**Fig 3:**
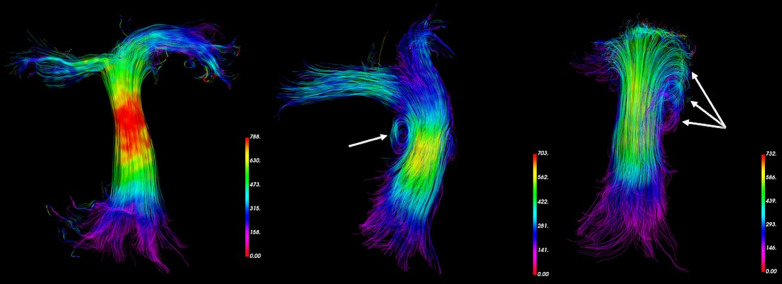
Examples of vortices observed through the PA streamlines (arrows), compared to a normal appearance (left panel). RHC mPAP was 22 mmHg, 30 mmHg and 60 mmHg from left to right panel, respectively. Bias (solid lines) and LOA (dashed lines) are displayed.

The median time between MRI and right heart catheterization was 3 days. Five patients did not present with PH at RHC and seventeen patients had myocardial septal fibrosis in late enhancement MRI sequences. The distribution of patients according to the international clinical classification of PH and the corresponding RHC measurements are summarized in Table 3. In patients with elevated pulmonary arterial pressure, vortical structures were observed in PA (Fig 3).

**Table 3:** Pulmonary pressures and resistances in RHC according to PH groups.

	sPAP	dPAP	mPAP	PVR
No PH (n = 4)	28.5 (5.9)	12 (3.9)	18.3 (4.3)	175.5 (35.5)
Group 1 PAH (n = 7)	66.7 (13.8)	25 (5.7)	40 (7.5)	478.6 (124.2)
Group 2 (n = 1)	47	21	30	365
Group 3 (n = 6)	52.5 (23.9)	26.5 (13.8)	35.8 (17.2)	515.5 (282.7)
Group 4 (n = 2)	67 (29.7)	27 (9.9)	40.5 (16.3)	438 (319.6)
Group 5 (n = 2)	59 (24)	18.5 (9.2)	32.5 (13.4)	470.5 (270.8)

Note: PH pulmonary hypertension, m/s/d PAP mean/systolic/diastolic pulmonary arterial pressure, PVR pulmonary vascular resistance.

### Comparison of MRI PA pressure estimation with RHC

Hemodynamic and morphological measurements from RHC and MRI are summarized in Table 4.

**Table 4:** Hemodynamic and morphological measurements from RHC and MRI.

Variables	Median (IQR)
**RHC**	
mPAP (mmHg)	32 (23-42)
sPAP (mmHg)	56 (34-72)
dPAP (mmHg)	20 (17-29)
RAP (mmHg)	6 (5-7)
PAWP (mmHg)	7 (5-8)
PVR (dynes.s.cm-5)	375.5 (212-612)
CO (L/min)	5.3 (4.3-6)
**MRI**	
Indexed RVEDV (mL/m²)	90.5 (67-130)
RV/LV from 4D flow	1.2 (1-1.3)
RVEF (%)	34 (28-47)
LVEF (%)	60 (51-66)
RV mass (g)	34 (27-46)
Relative change in PA cross-sectional area 4D	0.2 (0.1-0.2)

Note: *Values expressed as median (IQR)*, RHC right heart catheterization, MRI magnetic resonance imaging, m/s/d PAP mean/systolic/diastolic pulmonary arterial pressure, RAP right atrial pressure, PAWP pulmonary artery wedge pressure, CO cardiac output, PVR pulmonary vascular resistance. RV right ventricle, TVR tricuspid valve regurgitation, RV right ventricle, LV left ventricle, RVEF/LVEF right ventricle/left ventricle ejection fraction, PA pulmonary artery, RVEDV right ventricle end-diastolic volume.

mPAP and sPAP measurements from RHC were significantly correlated with multiple morphological and hemodynamic MRI parameters (Table 5). PA vorticity magnitude was significantly correlated with both mPAP and sPAP whereas helicity parameter was only correlated with sPAP.

**Table 5:** Correlations between PAPm or PAPs and MRI measurements.

	mPAP		sPAP	
	r	p	r	p
RVEDD	0.59	0.004*	0.57	0.010*
RVEDV	0.64	0.001*	0.62	0.002*
RVESV	0.61	0.003*	0.60	0.003*
RV mass	0.50	0.020*	0.52	0.010*
RV/LV diameters in 4D MRI	0.62	0.001*	0.61	0.003*
A_max_ PA4D	0.80	<0.001*	0.78	<0.001*
A_min_ PA 4D	0.79	<0.001*	0.77	<0.001*
Vorticity (90th percentile) within the PA	−0.51	0.020*	−0.53	0.010*
Maximum helicity within the RV	0.30	0.170	0.46	0.030*

Note: * p < 0.05, r correlation coefficient, m/s PAP mean/systolic pulmonary arterial pressure, RVEDD right ventricle end-diastolic diameter, RVEDV right ventricle end-diastolic volume, RVESV right ventricle end-systolic volume, RV right ventricle, LV left ventricle, MRI magnetic resonance imaging, PA pulmonary artery, Amax PA pulmonary artery maximum cross-sectional area, Amin PA pulmonary artery minimum cross-sectional area.

### Multivariate estimation models for mPAP and sPAP

The multivariate linear regression analysis resulted in the following catheterized mPAP and sPAP estimation models (Table 6):

**Table 6:** Multiple linear regression models.

Model variables	Coefficient	p
**mPAP**		
Constant value	− 2.420	0.690
A_max_ PA in 4D MRI	0.040	<0.001
Mean helicity RV	0.061	0.040
**sPAP**		
Constant value	23.980	0.080
A_max_ PA in 4D MRI	0.066	<0.001
Mean helicity RV	0.134	0.002
Mean vorticity in RA	−0.613	0.020

Note: m/s PAP: mean/systolic pulmonary arterial pressure, Amax PA: pulmonary artery maximum cross-sectional area, RA: right atrium, RV: right ventricle, MRI: magnetic resonance Imaging.

- **mPAP =** 0.04.A_max__PA_4D + 0.061.mean_helicity_RV – 2.42, with R² = 0.69 and adjusted R² = 0.66 (p = 0.04).
- **sPAP =** 0.066.A_max__PA_4D + 0.134.mean_helicity_RV – 0.613.mean_vorticity_RA + 23.98, with R² = 0.80 and adjusted R² = 0.76 (p = 0.02).

**Fig 4:**
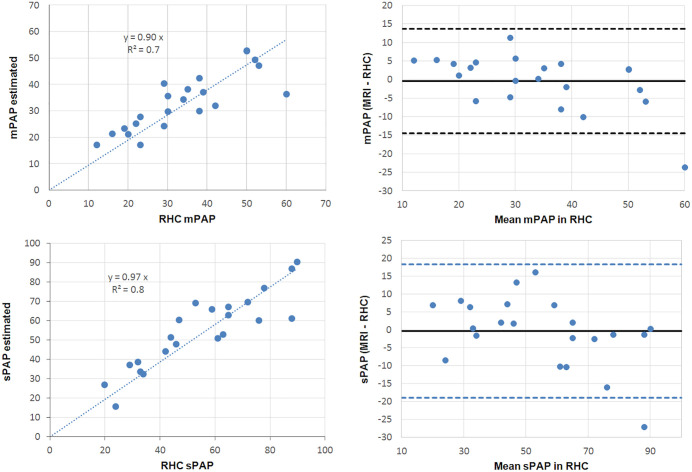
Scatter plots with regression lines and Bland-Altman plots for the comparison between MRI pressure estimations (mPAP and sPAP) and pulmonary arterial pressures measured via RHC.

The resulting scatter plots of mPAP and sPAP measured in cardiac catheterization as a function of mPAP and sPAP estimated by the MRI models as well as the corresponding Bland-Altman plots are depicted in Fig 4.

Bland-Altman analysis showed a bias of −0.42 mmHg for mPAP (lower and upper limits of agreement LOA: −14.5 and 13.66 mmHg, respectively) and a bias of −0.38 mmHg for sPAP (LOA: −19.02; 18.26 mmHg) (Table 8).

**Table 8:** Bland-Altman bootstrap analysis.

Bootstrap analysis		Estimate [95%CI]
PAPm_model vs PAPm	**Bias**	− 0.42 [−3.60; 2.34]
	**Lower LOA**	− 14.50 [−22.66; – 6.37]
	**Upper LOA**	13.66 [8.64; 18.19]
PAPs_model vs PAPs	**Bias**	− 0.38 [−4.46; 3.45]
	**Lower LOA**	− 19.02 [−28.02; – 9.64]
	**Upper LOA**	18.26 [11.83; 23.96]
PAPm_val vs PAPm	**Bias**	0.37 [−4.21; 4.30]
	**Lower LOA**	− 13.63 [−20.41; – 2.85]
	**Upper LOA**	14.38 [8.30; 17.82]
PAPs_val vs PAPs	**Bias**	− 0.35 [−8.73; 6.34]
	**Lower LOA**	− 25.27 [−39.39; – 0.83]
	**Upper LOA**	24.56 [13.00; 29.35]

Note: m/s/d PAP mean/systolic/diastolic pulmonary arterial pressure, CI confidence interval, LOA Limit of Agreement, val validation cohort.

The PRESS R-squared values were 0.62 and 0.71, for the first and second model, respectively, with these values exhibiting a high degree of consistency with the corresponding adjusted R² values.

### Validation cohort

The RHC measurements in patients from the validation group are summarized in Table 7.

**Table 7:** Hemodynamic and morphological measurements from RHC and MRI.

Parameters	median (IQR)
**RHC**	
mPAP (mmHg)	34 (23-40)
sPAP (mmHg)	54 (41-78)
dPAP (mmHg)	22 (14-26)
RAP (mmHg)	6.5 (5-13)
PAWP (mmHg)	8 (7-12)
PVR (dynes.s.cm-5)	92 (8.1-212)
CO (L/min)	5.1 (3.9-6)

Note: *Values expressed as median (IQR)*, RHC right heart catheterization, MRI magnetic resonance imaging, m/s/d PAP mean/systolic/diastolic pulmonary arterial pressure, RAP right atrial pressure, PAWP pulmonary artery wedge pressure, CO cardiac output, PVR pulmonary vascular resistance.

**Fig 5:**
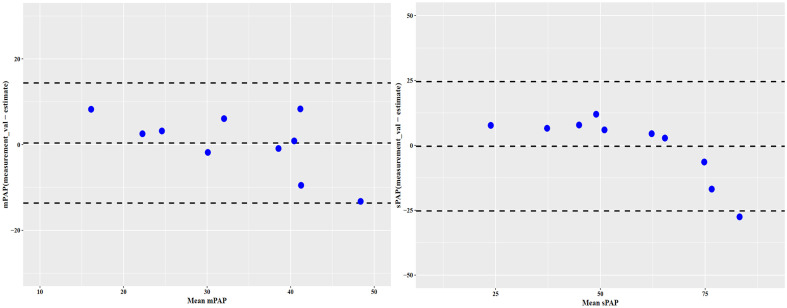
Bland-Altman plots comparing MRI pressure estimations (mPAP and sPAP) from the calculated model with pulmonary arterial pressures measured via RHC in the validation cohort.

The Bland–Altman plot (Fig 5) shows a relatively uniform distribution of differences across the measurement range, with no apparent trend or proportional bias.

All data points fell within the limits of agreement, supporting the consistency of the new method. The mean difference (bias) between the two methods was 0.37 and −0.35 units for mPAP and sPAP, respectively. These biases were not significantly different from zero (p = 0.87 and 0.93, respectively). The 95% limits of agreement ranged from −13.6 to 14.3 mmHg for mPAP and from −25.2 to 24.5 mmHg for sPAP (Table 8).

## Discussion

### Estimation of pulmonary artery pressure in MRI

In the present study, both mPAP and sPAP estimation models only included variables derived from 4D flow MRI, which seems sufficient for this task. The agreement between the pressures calculated from the MRI model and those measured during RHC was strong with respect to sample size. Our study population consisted of patients with suspected PH for whom cardiac catheterization was used as a diagnostic exam, but also of patients with known PH for whom cardiac catheterization was performed as part of the follow-up of the disease and re-evaluation under treatment. Furthermore, all categories of the clinical classification of PH were represented in our study. This relative heterogeneity strengthens the value of our PAP models as our population is representative of current clinical practice.

Both models included right ventricular helicity, which is consistent with the study by Schäfer al who showed that there was a significant difference between control and PH patients with respect to right ventricular outflow tract helicity [23]. As a complement to vorticity, helicity provides quantitative information on flow patterns. The pressure estimation models also included the maximum systolic PA cross-sectional area, representative of PA dilation, which increases during the evolution of the disease [5,23,32]. Johns et al. showed that a multivariate model including right ventricular mass, interventricular septal angle and pulmonary artery size in 102 COPD patients, 87 of whom had PH, exhibited a good diagnostic performance and a significant correlation with mPAP measured on right catheterization in the third group of PH classification (r = 0.732 and r² = 0.54) [32]. MRI and catheterization were performed within a maximum period of 90 days. The architectural changes that could occur in the RV and PA during a large delay between these exams must be considered when establishing the pressure estimation model from MRI. Nogami et al. showed a significant correlation between sPAP measured in RHC and sPAP calculated using the modified Bernoulli equation, similar to the echocardiographic approximation, by measuring Vmax TVR on 2D flow sequences and adding a constant 10 mmHg value for RAP [33]. Regarding univariate correlations performed prior to the multivariate linear regression models, several studies have demonstrated significant correlations between catheterized mPAP and RV functional indices such as RVTDV, RVTSV, RVEF and RV mass [19,32]. These results are consistent with our study where significant correlations between sPAP, mPAP and RVEDD, RVESD or RV mass were observed. We also observed a significant correlation between PAP and RV/LV diameters ratio, measured from 4D flow MRI, which is a prognostic factor related to right ventricular dysfunction in PH [19,34]. In addition, we observed a negative but not significant correlation between mPAP and the relative change in PA cross-sectional area from the 4D flow sequence. Moreover, diastolic and systolic PA cross-sectional areas were significantly correlated with increased mPAP and sPAP in both 2D and 4D MRI which is consistent with the observations of several authors [5,23,32].

### Vorticity and helicity

Vorticity and helicity are parameters that characterize the hemodynamic flow disturbances. Helicity can be thought of as a measurement of the coupling between the vortex and the main flow. The more the normal to the vortex is parallel to the main flow, the higher the helicity; conversely, if the axis of the vortex is perpendicular to the bulk flow, the value will be low. This distinguishes transverse vortices from longitudinal vortices; visually, these vortices resemble a whirlpool or a helix, respectively [35].

We observed a significant correlation between the vorticity within the PA and mPAP or sPAP, and between the maximum helicity within the RV and sPAP. Only a few studies calculated the helicity parameter but more data about the relationship between vorticity and mPAP is available. Using 4 parameters including peak systolic vorticity in the main and right pulmonary artery, Kheyfets et al. proposed a promising model for estimating PVR in a population of 22 subjects including 17 advanced PH subjects [18]. Other authors explored the potential occurrence of vortices along the PA and the correlation between its duration and mPAP [18,20,34]. In 145 subjects including 69 PH patients, Reiters et al. demonstrated a strong significant correlation between the catheter-derived mPAP and the relative visual vortex duration throughout the cardiac cycle using a temporal resolution of 89 ms, poorer than the resolution used in the present study [20]. An earlier study from the same authors showed a strong correlation between mPAP and this parameter (r = 0.94) with a Bland-Altman’s bias of −0.2 ± 7.0 mmHg [10]. Nevertheless, this subjective parameter appears to suffer from a lack of robustness as Kroeger et al did not demonstrate any significant correlation between vortex duration and mPAP in PH patients [22]. In the present study the vorticity and helicity parameters represent quantitative objective measurements.

### Limitations of the study

The main limitation of our study is the small sample size as well as its monocentric nature which limits the extrapolation of the results. Another limitation is related to the high encoding velocity and the spatio-temporal resolution of the 4D flow sequence which can lead to errors in the measurement of flow parameters [36]. A single VENC value was used for all chambers and vessels. Although optimized for the pulmonary artery, it may not have been ideal for evaluating the slower, more complex flow patterns within the RA and RV. Using lower VENC values or advanced techniques such as dual-VENC imaging could improve the assessment of intracavitary flow without compromising the assessment of high-velocity regions. In the present study, the temporal resolution of the 4D flow sequence (63 ms) was inferior to the recommended value set at 40 ms [37] whereas our spatial resolution agreed with the recommendations since the recommended resolution is < 2.5–3 mm [38]. However, our choice is the result of a compromise aiming at implementing a sequence compatible with the routine clinical practice. In addition, the examinations were performed in free breathing without respiratory gating, which reduced the acquisition time at the cost of a potential kinetic blur that could affect the quality of the images obtained. Finally, although a standardized preprocessing and analysis workflow was applied consistently across all subjects, no formal intra- or interobserver reproducibility analysis was performed.

In the future, other 4dflow derived parameters such as the specific PA vorticity circulation marker [39] or RV energetic parameters could be of interest in monitoring disease progression and therapeutic response [40]. Future studies should also incorporate time-resolved intracardiac pressure measurements with time-resolved 4D flow MRI–derived parameters. Such an approach would enable a dynamic coupling between pressure and flow organization across the cardiac cycle and may provide deeper mechanistic insights. Coupling the hemodynamic parameters with other biomarkers in large cohorts analyzed using artificial intelligence techniques might also improve management of PAH patients [41,42].

In summary, our study shows that an accelerated 4D flow MRI sequence appears to be a promising non-invasive tool for the estimation of pulmonary artery pressure in the diagnosis and follow-up of PH. Indeed, our models for estimating mPAP and sPAP, including only parameters derived from this sequence, correlate well with RHC pressure measurements. Moreover, the duration of the 4D flow sequence used in our study makes it relevant in routine clinical practice as it does not significantly extend the duration of the usual MRI protocol performed for PH assessment.

## Supporting information

S1 File Supplementary methods. (DOCX)

S2 File Supplementary figures. (PDF)

## Abbreviations

MRI: Magnetic Resonance Imaging
PH: Pulmonary hypertension
PA: Pulmonary artery
RHC: Right heart catheterization
m/s/d PAP: Mean/systolic/diastolic pulmonary arterial pressure
RV: Right ventricle
RA: Right atrium
Amax/min: PA maximum/minimum cross-sectional area
PVR: Pulmonary vascular resistance
V_max_ TVR: Maximal velocity of the tricuspid valve regurgitation
RAP: Right atrial pressure
LV: Left ventricle
PAWP: Pulmonary artery wedge pressure
CO: cardiac output
CI: cardiac index
bSSFP: balanced Steady-State Free Precession
RVEF/LVEF: Right ventricle/ Left ventricle ejection fraction
V_max_ PVR: Maximal velocity of pulmonary valvular regurgitation
LVEDD/ RVEDD: LV/ RV end-diastolic diameters
LVEDV/ RVEDV: Left/ right ventricle end-diastolic volume
LVESV/ RVESV: Left/ right ventricle end-systolic volume
V_max_ PA: Maximum PA trunk velocity
VIF: Variance inflation factor
LOA: Limits of agreement
COPD: Chronic obstructive pulmonary disease

## References

[1] Galiè N, McLaughlin VV, Rubin LJ, Simonneau G. An overview of the 6th World Symposium on Pulmonary Hypertension. Eur Respir J. 2019;53(1):1802148. doi: 10.1183/13993003.02148-2018 30552088 PMC6351332

[2] Galiè N, Humbert M, Vachiery J-L, Gibbs S, Lang I, Torbicki A, et al. 2015 ESC/ERS Guidelines for the diagnosis and treatment of pulmonary hypertension: The Joint Task Force for the Diagnosis and Treatment of Pulmonary Hypertension of the European Society of Cardiology (ESC) and the European Respiratory Society (ERS): Endorsed by: Association for European Paediatric and Congenital Cardiology (AEPC), International Society for Heart and Lung Transplantation (ISHLT). Eur Heart J. 2016;37(1):67–119. doi: 10.1093/eurheartj/ehv317 26320113

[3] Condon DF, Nickel NP, Anderson R, Mirza S, de Jesus Perez VA. The 6th World Symposium on Pulmonary Hypertension: what’s old is new. F1000Research. 2019;8:F1000 Faculty Rev-888. doi: 10.12688/f1000research.18811.1 PMC6584967 31249672

[4] Simonneau G, Montani D, Celermajer DS, Denton CP, Gatzoulis MA, Krowka M, et al. Haemodynamic definitions and updated clinical classification of pulmonary hypertension. Eur Respir J. 2019;53(1):1801913. doi: 10.1183/13993003.01913-2018 30545968 PMC6351336

[5] Sieren MM, Berlin C, Oechtering TH, Hunold P, Drömann D, Barkhausen J, et al. Comparison of 4D Flow MRI to 2D Flow MRI in the pulmonary arteries in healthy volunteers and patients with pulmonary hypertension. PLoS One. 2019;14(10):e0224121. doi: 10.1371/journal.pone.0224121 31648286 PMC6812822

[6] Hoeper MM, Lee SH, Voswinckel R, Palazzini M, Jais X, Marinelli A, et al. Complications of right heart catheterization procedures in patients with pulmonary hypertension in experienced centers. J Am Coll Cardiol. 2006;48(12):2546–52. doi: 10.1016/j.jacc.2006.07.061 17174196

[7] Reiter U, Reiter G, Kovacs G, Stalder AF, Gulsun MA, Greiser A, et al. Evaluation of elevated mean pulmonary arterial pressure based on magnetic resonance 4D velocity mapping: comparison of visualization techniques. PLoS One. 2013;8(12):e82212. doi: 10.1371/journal.pone.0082212 24349224 PMC3861394

[8] Fisher MR, Forfia PR, Chamera E, Housten-Harris T, Champion HC, Girgis RE, et al. Accuracy of Doppler echocardiography in the hemodynamic assessment of pulmonary hypertension. Am J Respir Crit Care Med. 2009;179(7):615–21. doi: 10.1164/rccm.200811-1691OC 19164700 PMC2720125

[9] Brennan JM, Blair JE, Goonewardena S, Ronan A, Shah D, Vasaiwala S, et al. Reappraisal of the use of inferior vena cava for estimating right atrial pressure. J Am Soc Echocardiogr. 2007;20(7):857–61. doi: 10.1016/j.echo.2007.01.005 17617312

[10] Reiter G, Reiter U, Kovacs G, Kainz B, Schmidt K, Maier R, et al. Magnetic resonance-derived 3-dimensional blood flow patterns in the main pulmonary artery as a marker of pulmonary hypertension and a measure of elevated mean pulmonary arterial pressure. Circ Cardiovasc Imaging. 2008;1(1):23–30. doi: 10.1161/CIRCIMAGING.108.780247 19808511

[11] Arcasoy SM, Christie JD, Ferrari VA, Sutton MSJ, Zisman DA, Blumenthal NP, et al. Echocardiographic assessment of pulmonary hypertension in patients with advanced lung disease. Am J Respir Crit Care Med. 2003;167(5):735–40. doi: 10.1164/rccm.200210-1130OC 12480614

[12] Freed BH, Collins JD, François CJ, Barker AJ, Cuttica MJ, Chesler NC, et al. MR and CT Imaging for the Evaluation of Pulmonary Hypertension. JACC Cardiovasc Imaging. 2016;9: 715–32. doi: 10.1016/j.jcmg.2015.12.015 27282439 PMC4905589

[13] Hur DJ, Sugeng L. Non-invasive Multimodality Cardiovascular Imaging of the Right Heart and Pulmonary Circulation in Pulmonary Hypertension. Front Cardiovasc Med. 2019;6:24. doi: 10.3389/fcvm.2019.00024 30931315 PMC6427926

[14] Swift AJ, Wild JM, Nagle SK, Roldán-Alzate A, François CJ, Fain S, et al. Quantitative magnetic resonance imaging of pulmonary hypertension: a practical approach to the current state of the art. J Thorac Imaging. 2014;29(2):68–79. doi: 10.1097/RTI.0000000000000079 24552882 PMC4015452

[15] Marrone G, Mamone G, Luca A, Vitulo P, Bertani A, Pilato M, et al. The role of 1.5T cardiac MRI in the diagnosis, prognosis and management of pulmonary arterial hypertension. Int J Cardiovasc Imaging. 2010;26(6):665–81. doi: 10.1007/s10554-010-9623-2 20336377

[16] Valdeolmillos E, Sakhi H, Tortigue M, Audié M, Isorni M-A, Lecerf F, et al. 4D flow cardiac MRI to assess pulmonary blood flow in patients with pulmonary arterial hypertension associated with congenital heart disease. Diagn Interv Imaging. 2024;105(7–8):266–72. doi: 10.1016/j.diii.2024.01.009 38368175

[17] Reiter U, Reiter G, Fuchsjäger M. MR phase-contrast imaging in pulmonary hypertension. Br J Radiol. 2016;89(1063):20150995. doi: 10.1259/bjr.20150995 26942293 PMC5257310

[18] Kheyfets VO, Schafer M, Podgorski CA, Schroeder JD, Browning J, Hertzberg J, et al. 4D magnetic resonance flow imaging for estimating pulmonary vascular resistance in pulmonary hypertension. J Magn Reson Imaging. 2016;44(4):914–22. doi: 10.1002/jmri.25251 27173445 PMC5331851

[19] Reiter U, Kovacs G, Reiter C, Kräuter C, Nizhnikava V, Fuchsjäger M, et al. MR 4D flow-based mean pulmonary arterial pressure tracking in pulmonary hypertension. Eur Radiol. 2021;31(4):1883–93. doi: 10.1007/s00330-020-07287-6 32974687 PMC7979582

[20] Reiter G, Reiter U, Kovacs G, Olschewski H, Fuchsjäger M. Blood flow vortices along the main pulmonary artery measured with MR imaging for diagnosis of pulmonary hypertension. Radiology. 2015;275(1):71–9. doi: 10.1148/radiol.14140849 25372980

[21] Ota H, Kamada H, Higuchi S, Takase K. Clinical Application of 4D Flow MR Imaging to Pulmonary Hypertension. Magn Reson Med Sci. 2022;21(2):309–18. doi: 10.2463/mrms.rev.2021-0111 35185084 PMC9680544

[22] Kroeger JR, Stackl M, Weiss K, Baeßler B, Gerhardt F, Rosenkranz S, et al. k-t accelerated multi-VENC 4D flow MRI improves vortex assessment in pulmonary hypertension. Eur J Radiol. 2021;145:110035. doi: 10.1016/j.ejrad.2021.110035 34801875

[23] Schäfer M, Barker AJ, Kheyfets V, Stenmark KR, Crapo J, Yeager ME, et al. Helicity and Vorticity of Pulmonary Arterial Flow in Patients With Pulmonary Hypertension: Quantitative Analysis of Flow Formations. J Am Heart Assoc. 2017;6(12):e007010. doi: 10.1161/JAHA.117.007010 29263034 PMC5779020

[24] Moffatt HK, Tsinober A. Helicity in Laminar and Turbulent Flow. Annu Rev Fluid Mech. 1992;24(1):281–312. doi: 10.1146/annurev.fl.24.010192.001433

[25] Saunders LC, Hughes PJC, Alabed S, Capener DJ, Marshall H, Vogel-Claussen J, et al. Integrated Cardiopulmonary MRI Assessment of Pulmonary Hypertension. J Magn Reson Imaging. 2022;55(3):633–52. doi: 10.1002/jmri.27849 34350655

[26] Assadi H, Uthayachandran B, Li R, Wardley J, Nyi TH, Grafton-Clarke C, et al. Kat-ARC accelerated 4D flow CMR: clinical validation for transvalvular flow and peak velocity assessment. Eur Radiol Exp. 2022;6(1):46. doi: 10.1186/s41747-022-00299-5 36131185 PMC9492816

[27] Lai P, Shimakawa A, Cheng JY, Alley MT, Vasanawala S, Brau AC. Sub-8-minute cardiac four dimensional flow MRI using kat ARC and variable density signal averaging. J Cardiovasc Magn Reson. 2015;17:1–3. doi: 10.1186/1532-429X-17-S1-Q3625589308

[28] Hirtler D, Garcia J, Barker AJ, Geiger J. Assessment of intracardiac flow and vorticity in the right heart of patients after repair of tetralogy of Fallot by flow-sensitive 4D MRI. Eur Radiol. 2016;26(10):3598–607. doi: 10.1007/s00330-015-4186-1 26747260 PMC4938791

[29] Efron B, Tibshirani RJ. An Introduction to the Bootstrap. CRC Press; 1994.

[30] Bland JM, Altman DG. Statistics notes: Bootstrap resampling methods. BMJ. 2015;350:h2622. doi: 10.1136/bmj.h2622 26037412

[31] Quan NT. The prediction sum of squares as a general measure for regression diagnostics. J Bus Econ Stat. 1988.

[32] Johns CS, Rajaram S, Capener DA, Oram C, Elliot C, Condliffe R, et al. Non-invasive methods for estimating mPAP in COPD using cardiovascular magnetic resonance imaging. Eur Radiol. 2018;28(4):1438–48. doi: 10.1007/s00330-017-5143-y 29147768 PMC5834560

[33] Nogami M, Ohno Y, Koyama H, Kono A, Takenaka D, Kataoka T, et al. Utility of phase contrast MR imaging for assessment of pulmonary flow and pressure estimation in patients with pulmonary hypertension: comparison with right heart catheterization and echocardiography. J Magn Reson Imaging. 2009;30(5):973–80. doi: 10.1002/jmri.21935 19856412

[34] Alabed S, Garg P, Johns CS, Alandejani F, Shahin Y, Dwivedi K, et al. Cardiac Magnetic Resonance in Pulmonary Hypertension-an Update. Curr Cardiovasc Imaging Rep. 2020;13(12):30. doi: 10.1007/s12410-020-09550-2 33184585 PMC7648000

[35] von Knobelsdorff-Brenkenhoff F, Trauzeddel RF, Barker AJ, Gruettner H, Markl M, Schulz-Menger J. Blood flow characteristics in the ascending aorta after aortic valve replacement--a pilot study using 4D-flow MRI. Int J Cardiol. 2014;170(3):426–33. doi: 10.1016/j.ijcard.2013.11.034 24315151 PMC5099073

[36] Stalder AF, Russe MF, Frydrychowicz A, Bock J, Hennig J, Markl M. Quantitative 2D and 3D phase contrast MRI: optimized analysis of blood flow and vessel wall parameters. Magn Reson Med. 2008;60(5):1218–31. doi: 10.1002/mrm.21778 18956416

[37] Dyverfeldt P, Bissell M, Barker AJ, Bolger AF, Carlhäll C-J, Ebbers T, et al. 4D flow cardiovascular magnetic resonance consensus statement. J Cardiovasc Magn Reson. 2015;17(1):72. doi: 10.1186/s12968-015-0174-5 26257141 PMC4530492

[38] Sträter A, Huber A, Rudolph J, Berndt M, Rasper M, Rummeny EJ. 4D-Flow MRI: Technique and Applications. ROFO Fortschr Geb Rontgenstr Nuklearmed. 2018;190:1025–35. doi: 10.1055/a-0647-2021 30103237

[39] Tsuchiya N, Nagao M, Shiina Y, Miyazaki S, Inai K, Murayama S, et al. Circulation derived from 4D flow MRI correlates with right ventricular dysfunction in patients with tetralogy of Fallot. Sci Rep. 2021;11(1):11623. doi: 10.1038/s41598-021-91125-2 34079023 PMC8172849

[40] Zhao X, Leng S, Tan R-S, Chai P, Yeo TJ, Bryant JA, et al. Right ventricular energetic biomarkers from 4D Flow CMR are associated with exertional capacity in pulmonary arterial hypertension. J Cardiovasc Magn Reson. 2022;24(1):61. doi: 10.1186/s12968-022-00896-8 36451198 PMC9714144

[41] Fadilah A, Putri VYS, Puling IMDR, Willyanto SE. Assessing the precision of machine learning for diagnosing pulmonary arterial hypertension: a systematic review and meta-analysis of diagnostic accuracy studies. Front Cardiovasc Med. 2024;11:1422327. doi: 10.3389/fcvm.2024.1422327 39257851 PMC11385608

[42] Han P-L, Jiang L, Cheng J-L, Shi K, Huang S, Jiang Y, et al. Artificial intelligence-assisted diagnosis of congenital heart disease and associated pulmonary arterial hypertension from chest radiographs: A multi-reader multi-case study. Eur J Radiol. 2024;171:111277. doi: 10.1016/j.ejrad.2023.111277 38160541

