# Supplementary material for: Quantification of pulmonary arterial pressure with 4D flow cardiac MRI velocity mapping in patients with suspected pulmonary hypertension: Comparison with right heart catheterization
Source: PLoS One. 2026 Apr 23;21(4):e0346600. doi: 10.1371/journal.pone.0346600 (PMC13105347; doi:10.1371/journal.pone.0346600)
Supplement: S1 File — (DOCX) [file pone.0346600.s001.docx]

**SUPPLEMENTARY DATA**

*** Supplementary Methods**

Post-processing of 4D flow images were performed using a dedicated in-house software specifically written in C++ language. This visualization and analysis software is based upon Qt (*Qt Company Ltd, https://www.qt.io/*) for the user interface and VTK/ITK libraries (Visualization and Insight toolkits, *Kitware Inc, https://vtk.org/ & https://itk.org/*) for 2D/3D rendering.

**Preprocessing:**

After importation of the DICOM series, images were cropped and a semi-automatic correction of phase offset was performed prior to further processing. This correction step involved a 2D third order polynomial model in a plane-by-plane basis.

As an option, an anti-aliasing algorithm could be performed over the whole volume of images at user's request. This algorithm was implemented using the ***ITKPhase Module*** from the ITK library.

**Streamlines:**

Streamlines representations were processed using the ***vtkStreamTracer*** class from the VTK visualization framework. The flow paths were calculated from seed points scattered around the center of mass of the vessel section of interest. Numerical integration was performed using a 5^th^ order Runge-Kutta algorithm with adaptative stepsize control available within the ***vtkStreamTracer*** class**.**

**Vorticity-Helicity calculations:**

Vorticity was calculated using the ***VtkCellDerivatives*** class from the VTK visualization framework. This filter computes the velocity vector derivatives throughout the volume; for each vector, the partial derivatives are calculated along each direction, yielding the derivative matrix **G**:

| ∂u_x_/∂x | ∂u_x_/∂y | ∂u_x_/∂z |
| --- | --- | --- |
| ∂u_y_/∂x | ∂u_y_/∂y | ∂u_y_/∂z |
| ∂u_z_/∂x | ∂u_z_/∂y | ∂u_z_/∂z |

- As the vorticity vector is defined as the curl of the velocity vector, it can be easily determined from the components of the matrix G:

$\vec{Vorticity}= \vec{\boldsymbol{\nabla}} \wedge\vec{\mathbf{V}} = \left( \begin{matrix} G_{23}-G_{32} \\ G_{31}-G_{13} \\ G_{12}-G_{21} \end{matrix} \right)$

- Helicity is the scalar calculated from the integration of the scalar product between velocity and vorticity over a section of fluid:

$$Helicity=\int\vec{\mathbf{V}}.( \vec{\boldsymbol{\nabla}} \wedge\vec{\mathbf{V}})$$

In our post-processing software vorticity and helicity are expressed in s^-1^ and cm^3^/s^2^, respectively. Helicity values are, in addition, scaled by a factor 100 for convenience.

After the volume calculation has been carried out, helicity and vorticity parameters were extracted from the vessel section of interest. Then, the modulus of the vorticity vector was calculated for all elements inside this cross-section.

**References:**

* *The Visualization Toolkit.* Fourth Edition*.* Schroeder Will, Martin Ken, Lorensen Bill, (2006).  Kitware Inc. ISBN 978-1-930934-19-1

* *The ITK Software Guide: Introduction and Development Guidelines*. Fourth Edition. Johnson, McCormick, Ibanez, (2015) published by Kitware Inc. ISBN: 9781-930934-27-6

* *ITK: enabling reproducible research and open science.* McCormick M, Liu X, Jomier J, Marion C, Ibanez L, (2014). Front Neuroinform. 8:13. doi:10.3389/fninf.2014.00013

* *N-Dimensional Phase Unwrapping*. Vigneault D, Wang W, Tee M, Bluemke D, Noble A. The Insight Journal. 2015 May. http://hdl.handle.net/10380/3516

* ITK/VTK source codes:

*https://github.com/InsightSoftwareConsortium/ITK*

*https://gitlab.kitware.com/vtk/vtk*
