# Supplementary material for: Quantification of pulmonary arterial pressure with 4D flow cardiac MRI velocity mapping in patients with suspected pulmonary hypertension: Comparison with right heart catheterization
Source: PLoS One. 2026 Apr 23;21(4):e0346600. doi: 10.1371/journal.pone.0346600 (PMC13105347; doi:10.1371/journal.pone.0346600)

Supplementary figure1. Visualization of vortical structures in the RA (A) and RV (B). The structures are depicted using streamlines, complemented by the corresponding velocity vectors shown in a plane perpendicular to the vortex axis.

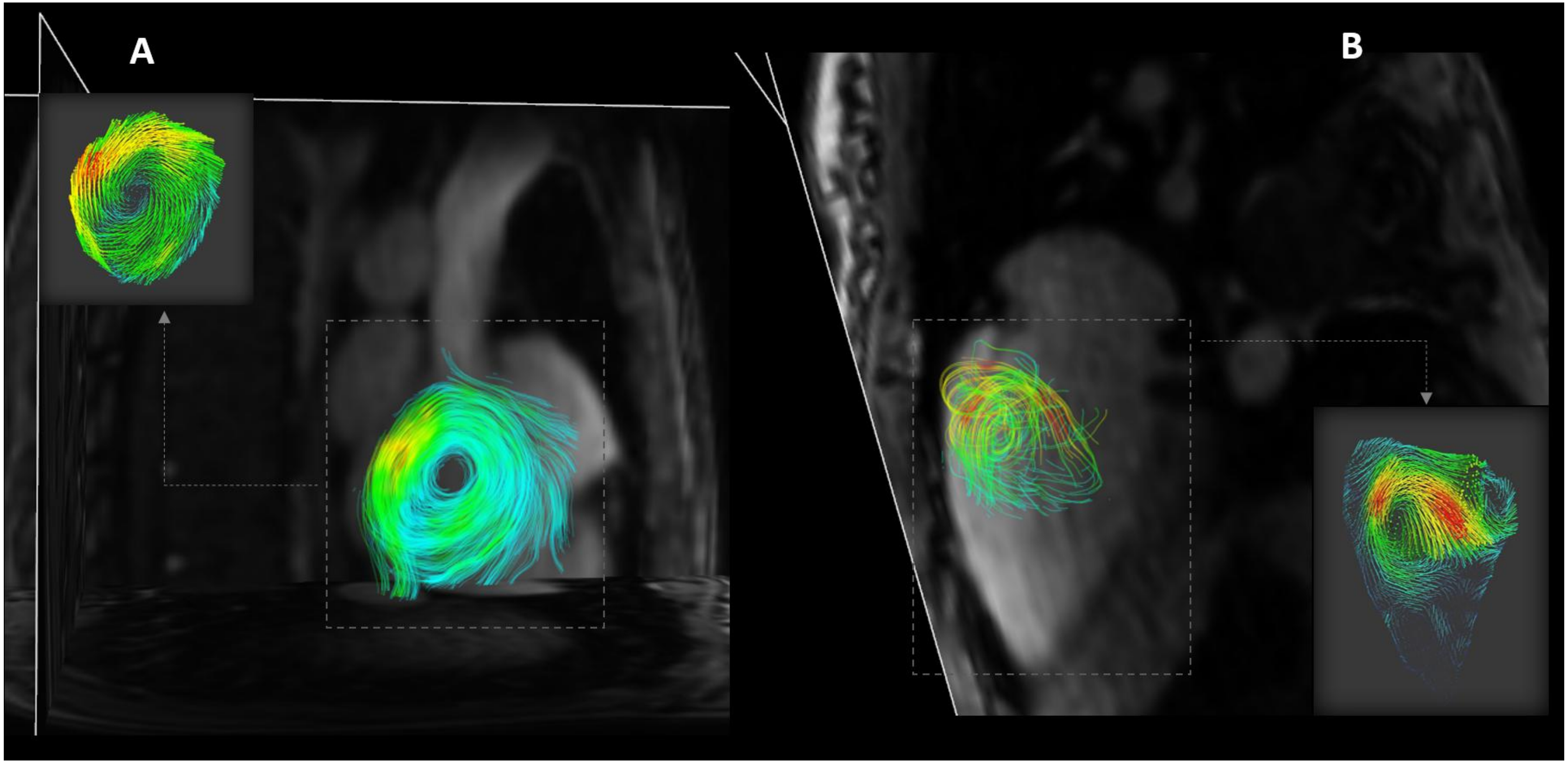

Supplement: S2 File — (PDF) [file pone.0346600.s002.pdf]
